# Perfluoropentane Phase-Change Nanodroplets for Focused Ultrasound-Enhanced Drug Penetration and Immune Response

**DOI:** 10.3390/pharmaceutics18030302

**Published:** 2026-02-27

**Authors:** Kichang Shin, Dongyeon Kim, Hyungwon Moon, Keunho Son, Mi Jeong Kim, Hak Jong Lee

**Affiliations:** 1 Department of Health Science and Technology, Graduate School of Convergence Science and Technology, Seoul National University, 1 Gwanak-ro, Gwanak-gu, Seoul 08826, Republic of Korea; skc5027@snu.ac.kr; 2 Department of Applied Bioengineering, Graduate School of Convergence Science and Technology, Seoul National University, 1 Gwanak-ro, Gwanak-gu, Seoul 08826, Republic of Korea; dongyeonk619@snu.ac.kr; 3 R&D Center, IMGT Co., Ltd., 172, Dolma-ro, Bundang-gu, Seongnam-si 13605, Republic of Korea; hyungwon.moon@nanoimgt.com (H.M.); keonho.son@nanoimgt.com (K.S.); 4 Department of Radiology, Seoul National University Bundang Hospital, Seongnam-si 13620, Republic of Korea; mijeong.kim@nanoimgt.com

**Keywords:** perfluoropentane nanodroplet, focused ultrasound, cavitation, acoustic droplet vaporization, drug delivery

## Abstract

**Background/Objectives**: Solid tumors are characterized by a dense stromal structure and heterogeneous microenvironments that limit intratumoral drug penetration and contribute to immune exclusion. We developed perfluoropentane (PFP)-based phase-change nanodroplets (IMP700) and aimed to identify focused ultrasound (FUS) parameters that enhance cavitation and sonoporation to improve drug delivery and immune engagement in tumor models. **Methods**: IMP700 was prepared as lipid-shelled PFP nanodroplets and physicochemically characterized. Acoustic droplet vaporization (ADV), echogenicity, and cavitation were evaluated in vitro and in vivo using ultrasound imaging and cavitation analysis under varying FUS parameters, including acoustic intensity, duty cycle, and pulse repetition frequency (PRF), in PANC-1 xenograft tumors. Sonoporation was assessed by co-administering an ultrasound-responsive doxorubicin liposome (IMP301), and intratumoral drug distribution was analyzed by confocal imaging. Immune responses were evaluated in a syngeneic 4T1 tumor model by quantifying CD8^+^ T-cell infiltration after repeated treatments. **Results**: IMP700 exhibited nanoscale size and high PFP encapsulation efficiency and underwent ADV with increased echogenicity and intensity-dependent cavitation. In vivo, a 2% duty cycle and 10 Hz PRF produced strong and reproducible cavitation. Under these conditions, IMP700 markedly increased inertial cavitation and enhanced intratumoral drug penetration compared to FUS alone. Combined IMP700 and FUS treatment also increased intratumoral CD8^+^ T-cell infiltration. **Conclusions**: IMP700 amplifies FUS-induced cavitation, improves sonoporation-mediated drug delivery, and promotes CD8^+^ T-cell infiltration, which supports the use of FUS-activated nanodroplets as a strategy to overcome stromal and immunological barriers in solid tumors.

## 1. Introduction

Physical modulation of the tumor microenvironment (TME) has emerged as a promising strategy to overcome therapeutic resistance in solid tumors by enhancing antitumor immune responses [1]. In immune-refractory tumors, physical and structural barriers within the TME restrict antigen accessibility, immune cell infiltration, and effective immune activation, which highlights the need for approaches capable of remodeling the tumor milieu in a spatially controlled manner. Among various physical modalities, ultrasound-based techniques offer unique advantages by enabling the noninvasive delivery of mechanical energy with high spatial precision [2].

In particular, acoustic cavitation, defined as the formation and dynamic oscillation of gas or vapor cavities in response to ultrasound exposure, is widely recognized as a central mechanism underlying the biological and immunologically relevant effects of ultrasound-based therapies [2,3]. Cavitation-induced mechanical stress has been shown to promote immunogenic signaling through enhanced tumor antigen release, the exposure of damage-associated molecular patterns, and the modulation of immune cell trafficking, thereby contributing to antitumor immune activation [4,5].

Focused ultrasound (FUS) enables cavitation activity to be spatially confined to a defined focal region within the tumor, allowing localized modulation of the tumor microenvironment without widespread tissue damage [6,7,8]. Depending on its spatial distribution, intensity, and temporal persistence, cavitation can induce a range of biological effects, including transient membrane permeabilization, microstructural remodeling, and localized mechanical stress, all of which are known to influence immune activation within tumors [9,10,11]. However, the reproducible induction and precise control of cavitation within the tumor parenchyma remain challenging, particularly in solid tumors with heterogeneous vascular architectures and stromal compositions, where cavitation patterns can vary substantially [12,13].

Importantly, emerging evidence indicates that the immunological consequences of cavitation are not solely determined by its magnitude but are also critically influenced by where cavitation occurs within the tumor microenvironment [14]. Cavitation localized predominantly to the vascular compartment may preferentially induce endothelial perturbation and transient permeability changes, whereas cavitation occurring within the tumor parenchyma can directly modulate the physical state of cancer cells and stromal components, thereby shaping antigen availability and immune accessibility [5,15]. This spatial distinction is particularly relevant in solid tumors, where immune-excluded and therapy-resistant regions—characterized by elevated interstitial pressure and dense extracellular matrix—are primarily located outside the vasculature [14].

Despite the recognized importance of cavitation localization for immune activation, most ultrasound-based strategies rely on microbubble-mediated cavitation that is largely confined to the intravascular compartment, due to the micron-scale size and vascular restriction of microbubbles, where acoustic conditions favor bubble activity [16,17]. While such approaches can robustly amplify cavitation signals, they provide limited control over cavitation events in extravascular regions, including the tumor interstitium and parenchyma, thereby restricting effective immune modulation within the tumor tissue. As a consequence, cavitation-induced immune responses are often spatially biased, transient, and difficult to reproduce across heterogeneous solid tumors [18,19].

Nanodroplets represent an emerging class of ultrasound-responsive platforms that offer a potential solution to this limitation [20]. Owing to their nanoscale dimensions, nanodroplets can extravasate beyond the vasculature and access tumor interstitial and parenchymal compartments, thereby repositioning cavitation nuclei within immune-relevant regions of the tumor [20]. Upon FUS exposure, nanodroplets undergo phase-transition–mediated activation through acoustic droplet vaporization (ADV), generating localized cavitation events directly within the tumor parenchyma [21]. This spatial redistribution of cavitation provides a means to couple precise physical targeting with reproducible immune activation.

**Figure 1:**
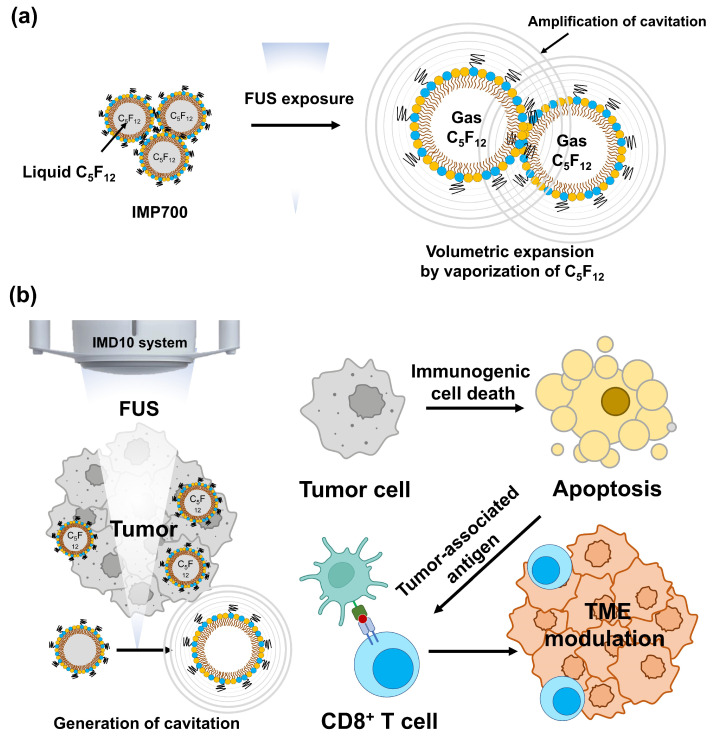
Schematic illustration of CD8^+^ T-cell infiltration mechanism. (**a**) Generation of cavitation by vaporization of C_5_F_12_ under FUS exposure, (**b**) mechanism of CD8^+^ T-cell infiltration. ICD: immunogenic cell death, TAA: tumor-associated antigen, TME: tumor microenvironment.

In this study, we developed perfluoropentane (PFP)-encapsulated lipid nanodroplets (IMP700) to investigate whether nanodroplet-assisted FUS can enhance antitumor immune responses through spatially controlled intratumoral cavitation. Using breast cancer and pancreatic cancer solid tumor models, we demonstrate that nanodroplets enable reproducible parenchymal cavitation patterns that are distinct from conventional vascular-confined responses, which leads to robust immune activation within the tumor microenvironment. Together, these findings establish controlled cavitation localization as a key physical determinant of ultrasound-mediated immune enhancement and provide a mechanistic framework for integrating focused ultrasound into immunotherapeutic strategies (Figure 1).

## 2. Materials and Methods

### 2.1. Materials

The following substances were acquired from Lipoid AG (Steinhausen, Switzerland): 1,2-Distearoyl-sn-glycero-3-phosphocholine (DSPC, purity: 99.9%), 1,2-Distearoyl-sn-glycero-3-phosphoethanolamine-N-[methoxy(polyethyleneglycol)-2000] (DSPE-mPEG2000, purity: 99.6%), 1,2-Dioleyl-sn-glycerol-3-phosphoethanolamine (DOPE, purity: 99.9%). 1-Stearoyl-2-lyso-sn-glycero-3-phosphocholine (S-LysoPC) was purchased from NOF Corporation (purity: >99.0%). Cholesterol (purity: 99.6%) was purchased from Sigma Aldrich (St. Louis, MO, USA). PFP was obtained from STREM Chemicals, Inc. (Newburyport, MA, USA), and IMP301 was acquired from IMGT. Co., Ltd. IMP301 is FUS-sensitive liposome [22]. Basic characteristics of IMP301 are demonstrated in Figure S1.

### 2.2. Preparation of Perfluoropentane-Encapsulated Lipid Nanodroplets, IMP700

IMP700s were prepared with the following lipid formation molar ratio: DSPC (40%), DSPE-mPEG2000 (9%), Cholesterol (6%), DOPE (35%), and S-LysoPC (10%). Firstly, all lipids were dissolved in chloroform, and the solvent was removed under a nitrogen stream using a rotary evaporator to form a dry lipid thin film. The resulting lipid films were hydrated in 0.01 M phosphate-buffered saline (PBS, pH 7.4) to a final concentration of 5 mg/mL by stirring at 500 rpm for 60 min at 50 °C. The mixture was stored under seal in the fridge to cool down to 4 °C, prior to the addition of PFP to a final concentration of 2% (*v*/*v* in PBS). Next, 1 mL of the mixture was placed in an 8 mL glass vial and sonicated using an ultrasonic device (UP200S, Hielscher Ultrasonics GmbH, Teltow, Germany) for 2 min to promote nanodroplet emulsion. The vial was in an ice bath to prevent temperature increase during sonication. Particle size homogenization was then achieved by extrusion through the 200 nm polycarbonate track-etch membranes (Cytiva, Marlborough, MA, USA) using a hand-driven mini-extruder (Avanti Polar Lipids, Inc., Alabaster, AL, USA).

### 2.3. Analysis of Size Distribution and the Number of IMP700s

The hydrodynamic diameter of the IMP700 was measured using a dynamic light scattering (DLS) apparatus (Zeta Sizer Nano-ZS, Malvern Instruments, Orsay, France) at a temperature of 25 °C. The IMP700s were diluted with 0.01 M phosphate-buffered saline (PBS). Three different samples for each batch were analyzed, and all DLS measurements were performed in triplicate. The hydrodynamic diameter is calculated as an average of 9 measurements and is reported as an intensity-based distribution using the cumulant method (Z-average). The concentration of IMP700s was also quantified using nanoparticle tracking analysis (NTA) on a Nanosight NS300 (Malvern Instruments, Malvern, UK). Before measurement, samples were gently vortexed and diluted to 1:1000 *v*/*v* using PBS (pH 7.4). Three independent samples were analyzed at 25 °C, with five 60 s videos recorded per sample.

### 2.4. Morphology and C_5_F_12_ Encapsulation Efficiency of IMP700

Cryogenic transmission electron microscopy (cryo-TEM) was performed to visualize the morphological changes in IMP700 nanoparticles before and after vaporization. For cryo-TEM analysis, samples were prepared using a Vitrobot-type plunge freezer (FEI FP5350/60). A 5 μL aliquot of the liposome suspension was applied to a carbon-coated TEM grid (Lacey support film, 200 mesh; Ted Pella, Inc., Redding, CA, USA), blotted with filter paper, and rapidly vitrified by plunging into liquid ethane. For analysis of the vaporized morphology, IMP700 was dropwise applied to TEM grids and vaporized in situ. The IMP700 was analyzed under cryogenic conditions at −170 °C using a Tecnai F20 X TXIN microscope (FEI, Hillsboro, OR, USA) equipped with a field emission gun operating at an acceleration voltage of 200 kV.

For the investigation of C_5_F_12_ encapsulation efficiency, gas chromatography was applied. The IMP700 was diluted by ethanol at a concentration of 100 µg/mL in a headspace vial. The diluted IMP700 was sonicated in a bath sonicator at 37 °C for 5 min to break all nanodroplets and, thus, release the C_5_F_12_. Prior to quantification, PFP in the headspace vial was fully evaporated by increasing the temperature in the headspace vial oven to 85 °C. Then, the PFP was evaluated by gas chromatography (GC) equipped with a flame ionization detector (FID). The temperature for PFP detection was maintained at 250 °C, and the flow rate of nitrogen gas was 5 mL/min. This process was repeated in triplicate for each formulation, and isopropyl alcohol was used as an internal standard.

### 2.5. Stability of IMP700

The stability of IMP700s over storage time was evaluated in 0.01 M PBS at storage temperature (4 °C). Changes in hydrodynamic size and concentration were monitored at predetermined time points (0, 1, 3, 6, 9, and 24 h) using DLS and NTA, respectively. To assess the temperature-dependent stability of IMP700s, IMP700s were placed in the refrigerator (4 °C) and on a temperature-controlled hot plate (37 °C and 60 °C). The morphological changes and evaporation of PFP were observed with an optical microscope, and the microscopic images were captured to evaluate the thermal stability of the IMP700.

### 2.6. Vaporization of IMP700 Dependent on the FUS Intensity

To investigate cavitation generation induced by vaporization of IMP700 under FUS exposure, IMP700 was irradiated with FUS at varying acoustic intensities. The FUS system was equipped with a FUS transducer operating at a center frequency of 1.1 MHz with a cigar-shaped acoustic focal zone measuring 1.2 mm in diameter and 9.4 mm in length at −6 dB. Briefly, IMP700 diluted 100-fold in PBS was loaded into a latex tube to minimize acoustic impedance mismatch. FUS was applied to the center of the latex tube, and ultrasound images were acquired during FUS irradiation. FUS was applied with intensities ranging from 0.35 to 2.81 kW/cm^2^, at a 5% duty cycle, with PRF of 10 Hz and 5 s irradiation duration, to investigate intensity-dependent ADV. During FUS exposure, real-time B-mode ultrasound images were acquired using the diagnostic imaging probe. The echogenicity of vaporized IMP700 droplets was quantified as the mean gray value within the ROI using ImageJ software (Version 1.54g) to assess the ultrasound-induced phase transition effect.

### 2.7. Cavitation Behavior of IMP700 as a Function of FUS Intensity

Cavitation dose was assessed across 0.5–2.5 kW/cm^2^, while the duty cycle and PRF were fixed at 2% and 10 Hz, respectively, to evaluate the cavitation behavior of IMP700 under varying ultrasound intensities. Stable and inertial cavitation levels were quantified from the same cavitation signals.

**Figure 2:**
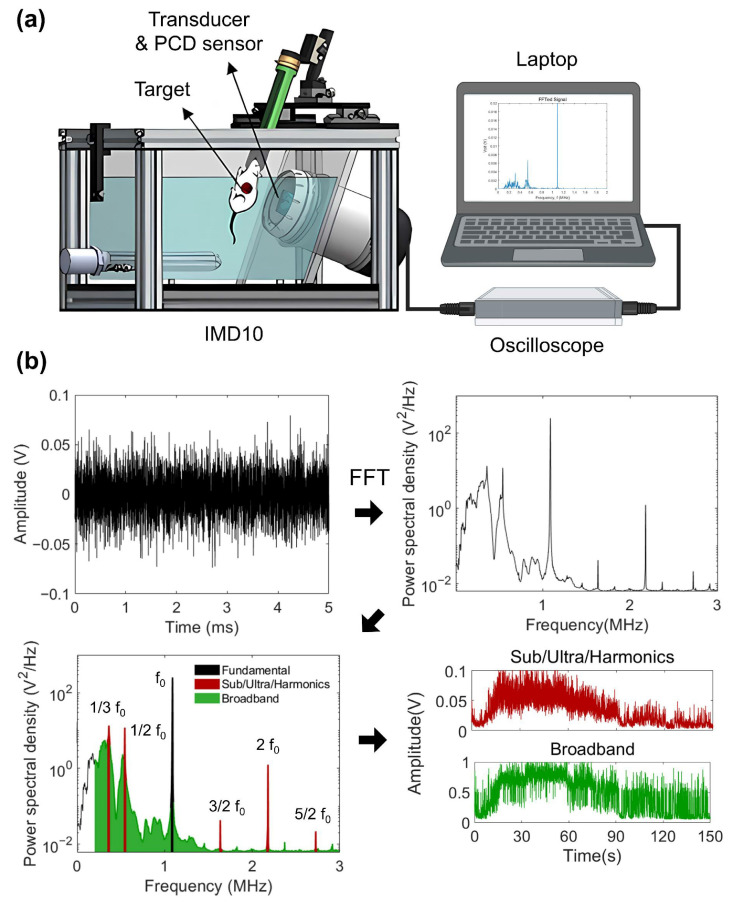
Schematic illustration of cavitation signal acquisition and analysis. (**a**) Cavitation signals were collected using the IMD10 system equipped with confocally aligned PCD sensors and transmitted to an oscilloscope connected to a laptop for signal recording and processing (created with BioRender.com). (**b**) Representative workflow for cavitation signal analysis. Time-domain signals were converted to frequency-domain spectra via FFT, and the PSD was used to extract frequency components corresponding to stable and inertial cavitation.

The overall process of cavitation signal acquisition and analysis is illustrated in Figure 2. Cavitation was measured using an IMD10 system (IMGT Co., Ltd., Seongnam-si, Republic of Korea) equipped with a FUS transducer and a pair of 6 mm diameter passive cavitation detection (PCD) sensors confocally aligned with the FUS focal point. The PCD sensors converted the acoustic signals into voltage signals, and the signals were measured by an oscilloscope device (NI 779969-1, National Instrument, Austin, TX, USA) and processed by the MATLAB software (Ver R2023a, MathWorks, Natick, MA, USA) (Figure 2a). Time-domain signals were converted into frequency-domain signals by a Fast Fourier transform (FFT). The power spectral density (PSD) was calculated to quantify the cavitation dose (Figure 2b).

Stable and inertial cavitation doses were quantified by integrating PSD over predefined spectral bands. The center frequency of the FUS transducer (f_c_ = 1.1 MHz) and subharmonic, ultraharmonic, and harmonic components were used to define stable cavitation bands [23,24,25,26]. By contrast, inertial cavitation was quantified from broadband except for the stable cavitation frequency [25,26,27], which encompasses all frequency regions (0.2–3 MHz), excluding the fundamental and stable cavitation bands. The frequency bands used for cavitation analysis are summarized in Table 1.

### 2.8. Mouse Tumor Modeling

PANC-1 human pancreatic cancer cells (ATCC, Manassas, VA, USA) and 4T1 murine mammary carcinoma cells (ATCC, Manassas, VA, USA) were grown in DMEM and RPMI 1640 with glutamine, respectively, supplemented with 10% fetal bovine serum (FBS), 100 U/mL penicillin, and 100 μg/mL streptomycin. Cells were cultured in a 5% CO_2_ humidified atmosphere at 37 °C. Both cells were harvested at 70–80% confluency and washed with PBS. PANC-1 cells were resuspended in PBS and Matrigel basement membrane matrix (Corning, NY, USA) mixture, while 4T1 cells were resuspended in PBS for tumor implantation.

For the PANC-1 xenograft model, female BALB/c nude mice (6–7 weeks old, 18–20 g of body weight) were subcutaneously inoculated in the right thigh with 5 × 10^6^ PANC-1 cells suspended in 50 μL PBS mixed with 50 μL Matrigel. For the syngeneic 4T1 model, female BALB/c mice (6–7 weeks old, 18–20 g of body weight) were anesthetized by intraperitoneal injection of a mixture of Zoletil (30 mg/kg; Virbac, Carros, France) and Rompun (10 mg/kg; Bayer, Leverkusen, Germany), and the hair on the right thigh was removed before tumor implantation. Then, 4T1 cells were subcutaneously injected into the right thigh at a density of 1 × 10^6^ cells in 100 μL PBS.

Tumor growth was monitored every 2–3 days using digital calipers, and tumor volume was calculated using the formula: V = (length × width × height)/2. Sonoporation and immune response experiments were initiated when tumor volumes reached approximately 150–200mm^3^. All animal experiments were performed following a protocol approved by the Institutional Animal Care and Use Committee (IACUC No. BA-2111-332-009-01, approved on 1 February 2023) of the Biomedical Research Institute of Seoul National University Bundang Hospital.

### 2.9. In Vivo IMP700 Vaporization and Cavitation Assessment Depending on the FUS Parameter

In vivo vaporization and cavitation of IMP700 were assessed using a PANC-1 xenografted mouse model. The vaporization efficiency of IMP700 was quantified by measuring echogenicity in ultrasound images using ImageJ software. To investigate the in vivo cavitation dose of IMP700 during vaporization, PANC-1 xenografted mice (*n* = 5 per group) were intravenously injected with 1–3 × 10^9^ droplets per mouse. The duty cycle was adjusted to 1%, 2%, 3%, and 5%, while the PRF was set to 10 Hz and 250 Hz. For each condition, FUS was applied to the tumor region, and the acoustic signals were recorded and analyzed to quantify the cavitation dose.

### 2.10. Sonoporation Effect of IMP700-Mediated Focused Ultrasound

Sonoporation efficiency was assessed using IMP301. A total of twenty PANC-1 xenografted mice were randomly divided into four groups, with five mice in each group: (1) vehicle-only control, (2) IMP301, (3) IMP301 with FUS, and (4) IMP301 + IMP700 with FUS. IMP301 (10 mg/kg) and IMP700 (1–3 × 10^9^ droplets in PBS) were intravenously co-administered at a total injection volume of 200 μL. After injection, FUS (2 kW/cm^2^, 2% duty cycle, and 10 Hz PRF) was applied, and the cavitation dose was measured. After FUS exposure, tumor tissues were harvested 1 h after treatment. The tumors were embedded and sectioned for fluorescence imaging. The tissue slices were mounted with DAPI-containing medium (Fluoroshield with DAPI, Immuno BioScience, Mukilteo, WA, USA) to stain the nuclei, and confocal fluorescence images were acquired using an LSM800 confocal laser scanning microscope (Carl Zeiss AG, Jena, Germany) to evaluate the intracellular uptake and distribution of IMP301.

### 2.11. Investigation of Immune Response Enhancement by Combining IMP700 and FUS Treatment

The enhancement of immune responses was evaluated using a syngeneic mouse model. The 4T1 syngeneic mouse was exposed to FUS with 2.5 kW/cm^2^ of intensity, 2%-duty cycle, and 10 Hz for 10s/exposure spot. The experimental groups were divided into a non-FUS-treated group (control), a FUS-treated group (FUS), and a combined IMP700 and FUS treatment group (IMP700 & FUS), with *n* = 3 per group. The FUS or IMP700 treatments were administered three times in 3-day intervals. Post 2 days of the 3rd FUS treatment, the tumor was excised for the CD8^+^ T-cell infiltration by immunohistochemistry analysis.

### 2.12. Immunohistochemistry

Tumor tissues were rinsed with PBS (pH 7.4) and fixed in 4% paraformaldehyde overnight. The tumors were paraffin-sectioned for immunostaining CD8α mouse antibody. Tumor slides were incubated with anti-mouse CD8α monoclonal rat antibody (Invitrogen (Carlsbad, CA, USA), 14-0808-82), as a primary antibody, at a 1:50 dilution (in normal horse serum solution) at 4 °C overnight. After washing with PBS, detection was carried out using a biotinylated anti-rat antibody (Vector Laboratories (Newark, CA, USA), BP-9400-50, host: Goat) and ABC solution kit (Vector Laboratories, PK-6100). Samples were incubated with secondary antibody and ABC solution for 1 h each at room temperature. Visualization was performed using diaminobenzidine (DAB) chromogen, followed by hematoxylin counterstaining before dehydration and coverslipping for imaging and quantitative analysis. After the dehydration step and the clearing step with xylene, the paramount solution was dropped on the slides, and the coverslipped slides were left overnight in the fume hood for xylene evaporation. The CD8α-stained tumor region was visualized by Pannoramic 250 Flash III digital whole-slide scanner (3DHISTECH Ltd., Budapest, Hungary), equipped with a Zeiss Plan-Apochromat 20×/0.8 NA objective lens. The CD8^+^ T-cell infiltration was quantified by measuring the stained area using the Quant Center image analysis platform (3D HISTECH).

### 2.13. Statistical Analysis

All data are expressed as average ± standard deviation. Statistical significance of the difference between the individual groups was determined by either a one-way or two-way ANOVA followed by Tukey’s HSD test or Welch’s ANOVA followed by Dunnett’s T3 test, depending on the homogeneity of variances. Statistical significance was defined as *p* < 0.05. Data analysis was performed using GraphPad Prism (version 10.6.1; GraphPad Software, San Diego, CA, USA).

## 3. Results

### 3.1. Size Distribution and Concentration of IMP700

**Figure 3:**
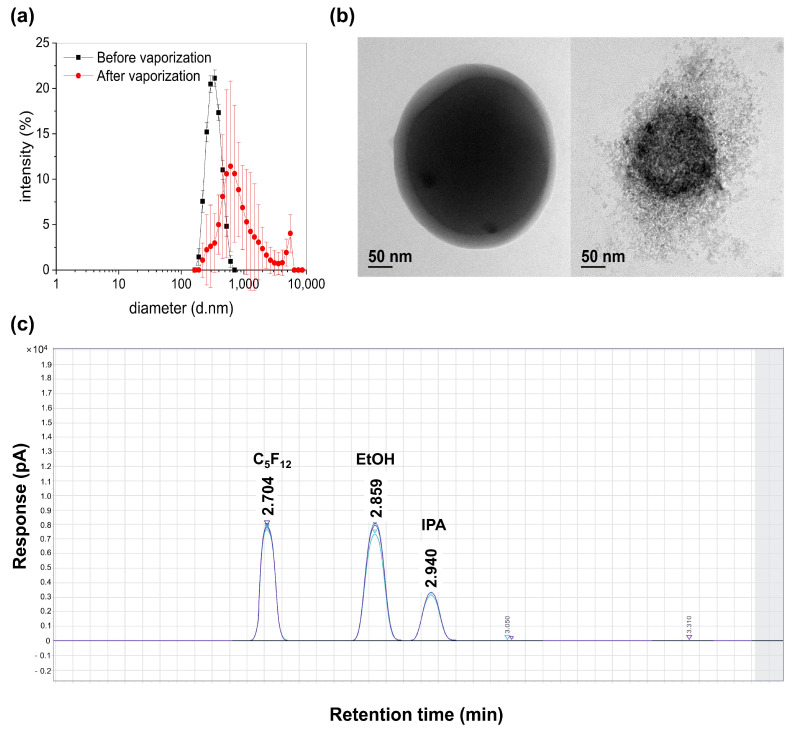
Physicochemical and structural characterization of C_5_F_12_-loaded lipid nanodroplets (IMP700). (**a**) Dynamic light scattering profiles of IMP700 before and after acoustic vaporization, showing a size shift from the nanoscale to the microscale range following phase transition. (**b**) Representative cryo-TEM images of IMP700 before (left) and after (right) vaporization, confirming the morphological transition from a compact, spherical droplet to a porous, gas-filled structure. (**c**) Gas chromatogram of the C_5_F_12_ core in IMP700, demonstrating the distinct retention peaks of C_5_F_12_, ethanol (EtOH), and isopropanol (IPA), *n* = 3.

Dynamic light scattering analysis revealed that the IMP700 had a Z-average diameter of 326.51 ± 64.73 nm and polydisperse index of 0.21 before vaporization, displaying a narrow nanoscale distribution (Figure 3a). Consistently, NTA measured a mean particle diameter of 285.2 ± 2.6 nm, further confirming the nanoscale size distribution of IMP700 (Figure S2a). Also, the particle size remained stable in the cell culture medium (DMEM), showing no significant shift compared to PBS (Figure S2b). Following FUS exposure, the size distribution shifted toward larger diameters, with a Z-average of 712.25 ± 563.78 nm, with the large polydisperse index value of 0.851 confirming successful vaporization and bubble expansion. The average concentration of IMP700 was measured to be 2.7 ± 0.2 × 10^11^ droplets/mL.

### 3.2. Morphology Analysis by Cryo-TEM Image

In Figure 3b, the cryo-TEM images of IMP700 before and after vaporization are shown. Before vaporization, the IMP700 exhibited a spherical morphology indicating stable droplet formation. However, after FUS irradiation, the IMP700 appeared disrupted and transformed into a heterogeneous porous structure, consistent with gas-filled bubble expansion and shell fragmentation. These observations confirm successful ADV and demonstrate structural destabilization consistent with liquid-to-gas phase transition.

### 3.3. C_5_F_12_ Encapsulation Efficiency

The chromatogram in Figure 3c clearly shows well-resolved peaks corresponding to PFP, ethanol, and isopropanol, with low peak dispersion and excellent reproducibility (% relative standard deviation, RSD = 1.17% for PFP). The PFP peak exhibited a sharp and distinct profile, indicating consistent formulation quality. Based on the quantified free PFP fraction relative to the total input amount, the encapsulation efficiency was calculated to be 99.5%, demonstrating that nearly all the incorporated PFP was successfully encapsulated within the nanodroplets.

### 3.4. Stability Analysis of IMP700 Dependent on the Time and Temperature

**Figure 4:**
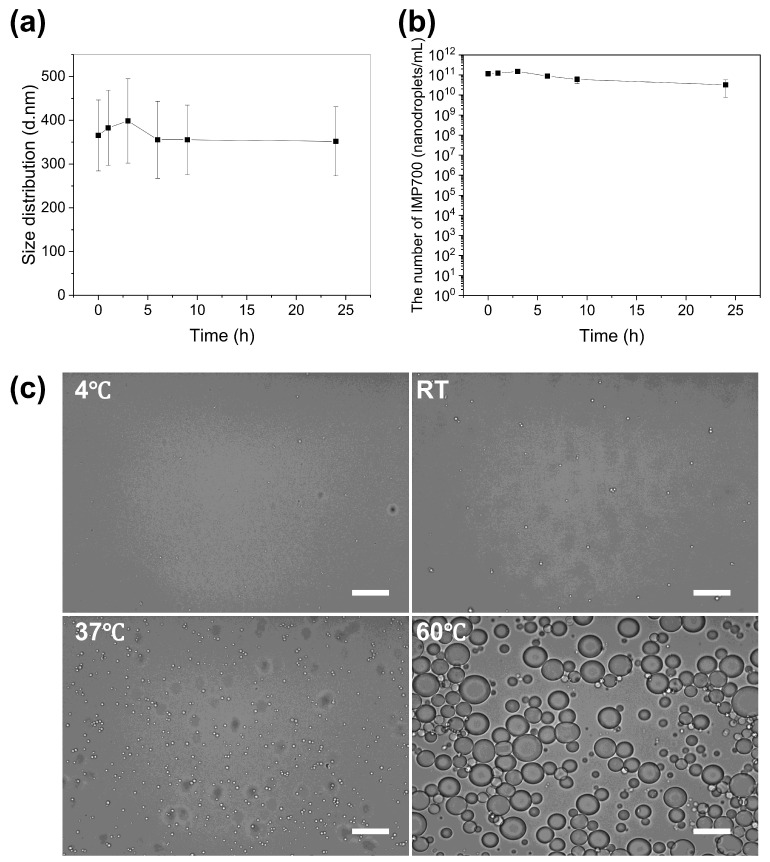
The stability of IMP700 under different storage times and temperatures. (**a**) Time-dependent change in the mean particle size of IMP700 over 24 h. (**b**) Number of IMP700 droplets measured over the same period. (**c**) Optical microscopy images of IMP700 at different temperatures (4 °C, room temperature, 37 °C, and 60 °C), showing temperature-dependent morphological changes. Scale bar, 20 µm.

The stability of IMP700 was evaluated as a function of storage time and temperature. As shown in Figure 4a, the mean particle size of IMP700 remained stable for up to 24 h, which confirms that the formulation retained its structural integrity, and the particle concentration was stably maintained for 24 h (Figure 4b).

Microscopic observation under different temperature conditions further demonstrated distinct morphological changes (Figure 4c). At 4 °C, IMP700 particles maintained their spherical morphology without noticeable structural changes, and only a few microbubbles (MBs) were visible. At room temperature, several IMP700s became visibly larger under microscopic observation. As the temperature increased to 37 °C, a greater proportion of IMP700s expanded and transformed into visible MBs. When heated to 60 °C, extensive bubble formation occurred, and the MBs reached diameters exceeding 10 µm, which indicates that the IMP700 underwent a complete phase transition and consequently lost its structural integrity.

### 3.5. In Vitro Ultrasound Echogenicity After Acoustic Droplet Vaporization

**Figure 5:**
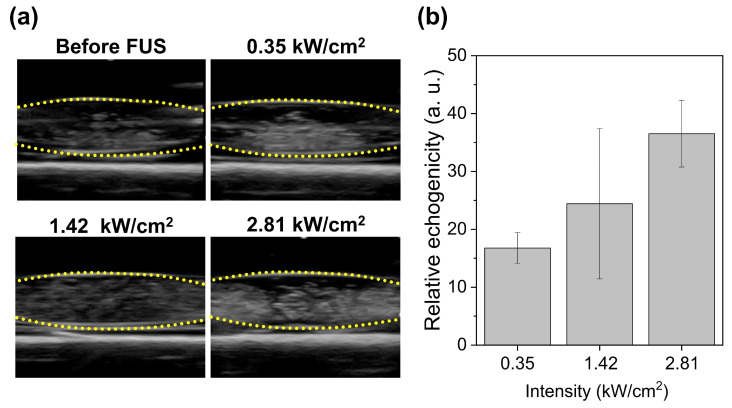
Echogenicity of IMP700 nanodroplets under focused ultrasound (FUS) irradiation. (**a**) Representative B-mode ultrasound images of IMP700 before FUS exposure and after irradiation at different acoustic intensities (0.35, 1.42, and 2.81 kW/cm^2^), showing enhanced echogenicity corresponding to vaporization of the nanodroplets. Yellow dotted lines indicate the region of interest. (**b**) Quantitative analysis of relative echogenicity (mean gray value, a.u.) as a function of FUS intensity (1 MHz, 5% duty cycle, 10 Hz PRF, 5 s exposure per spot).

FUS irradiation led to a noticeable increase in the echogenicity of IMP700 in the latex tube, which demonstrates the effective vaporization of IMP700 under in vitro conditions (Figure 5a). As shown in Figure 5b, the echogenic response intensified progressively as the applied acoustic intensity increased, with a mean gray value of 46.94 ± 2.64, 54.59 ± 12.98, and 66.7 ± 5.76 (a.u.) at 0.35, 1.42, and 2.81 kW/cm^2^, respectively. These findings demonstrate that IMP700 undergoes vaporization under FUS and that the degree of vaporization is governed by the intensity of FUS.

### 3.6. In Vitro Cavitation Measurement

**Figure 6:**
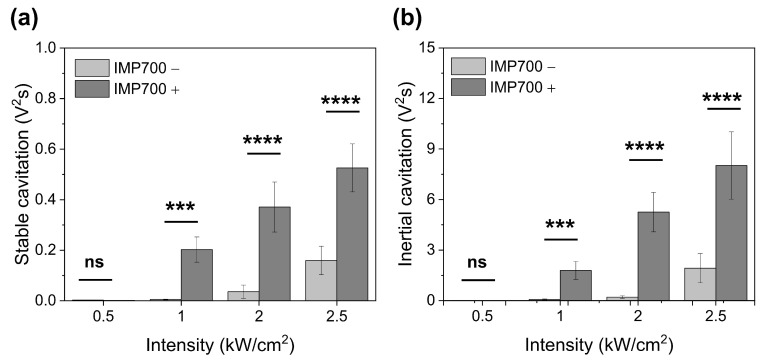
Cavitation behavior of IMP700 nanodroplets under FUS irradiation. (**a**) Stable cavitation dose and (**b**) inertial cavitation dose measured at different acoustic intensities (0.5, 1, 2, and 2.5 kW/cm^2^) with or without nanodroplets. Statistical analysis was performed using two-way ANOVA with Bonferroni’s post hoc test; ns, not significant; *** *p* < 0.001, **** *p* < 0.0001.

The IMP700 showed increased acoustic cavitation with rising ultrasound intensity (Figure 6). In the stable cavitation measurements, IMP700 produced minimal activity at 0.5 kW/cm^2^, followed by a clear increase to 0.2 V^2^s at 1.0 kW/cm^2^. The cavitation dose continued to rise to 0.371 V^2^s and 0.526 V^2^s at 2.0 and 2.5 kW/cm^2^, respectively, and remained consistently higher than that of FUS alone across all intensities (Figure 6a). A similar pattern was observed for inertial cavitation (Figure 6b).

Inertial cavitation was negligible at 0.5 kW/cm^2^, having a cavitation dose less than 0.01 V^2^s, but increased sharply to 1.78V^2^s at 1.0 kW/cm^2^ and 5.25 V^2^s at 2.0 kW/cm^2^ in the presence of IMP700, with a further rise at 2.5 kW/cm^2^, having a cavitation dose greater than 8 V^2^s. Overall, IMP700 yielded stronger cavitation signals than FUS alone, and 2.0 kW/cm^2^ was selected for further experiments.

### 3.7. In Vivo Assessment of IMP700 Vaporization Relative to the FUS Intensity by US Imaging

**Figure 7:**
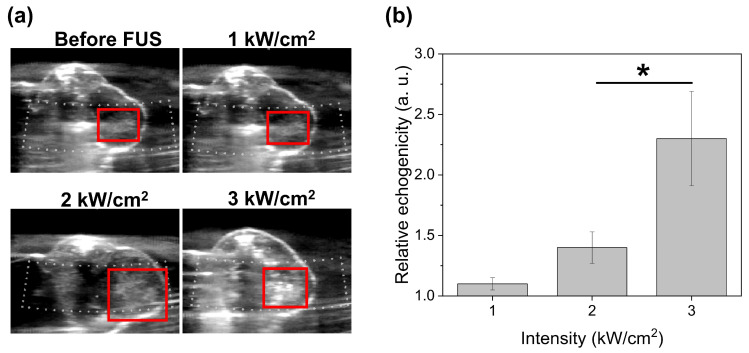
Echogenicity of IMP700 in PANC-1 xenografted mouse after FUS irradiation with intensities of 1, 2, and 3 kW/cm^2^. (**a**) Ultrasound image of PANC-1 xenografted mouse, (**b**) quantitative analysis of echogenicity at tumor region (red box). * *p* < 0.05.

To evaluate whether IMP700 underwent vaporization upon FUS exposure, a PANC-1 tumor model was established and treated under real-time US image guidance. As shown in Figure 7a, the echogenic response of IMP700 was assessed following FUS irradiation. In the absence of FUS exposure, the tumor region exhibited no appreciable contrast enhancement after IMP700 administration, which indicates the minimal intrinsic echogenicity of the formulation. Similarly, FUS treatment at 1.0 kW/cm^2^ resulted in a slight increase in contrast intensity within the tumor region (1.1 ± 0.05-fold) compared to the non-FUS-irradiated control. By contrast, the effective enhancement of both the gray-scale contrast area and mean gray value was observed at higher acoustic intensities. Specifically, FUS irradiation at 2.0 and 3.0 kW/cm^2^ led to significant increases in contrast intensity by 1.4 ± 0.13-fold and 2.3 ± 0.39-fold, respectively (Figure 7b). These results suggest that IMP700 undergoes efficient phase transition under sufficiently high acoustic pressure, which results in increased echogenicity that is detectable by US imaging.

### 3.8. In Vivo Cavitation Dose Depending on the FUS Irradiation

**Figure 8:**
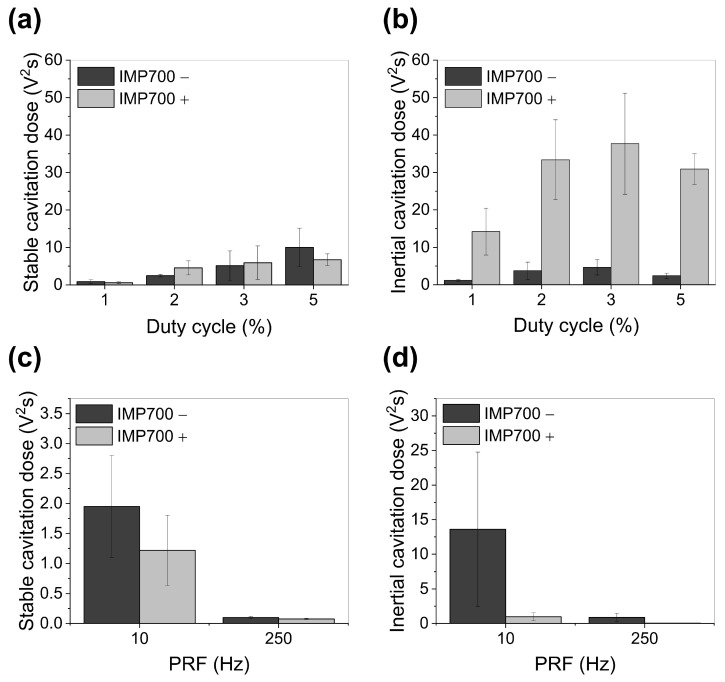
Cavitation dose at the tumor region in PANC-1 xenografted mouse after intravenous administration of IMP700. (**a**) Stable and (**b**) inertial cavitation dose at the tumor region with 1, 2, 3, and 5% of duty cycle. (**c**) Stable and (**d**) inertial cavitation dose at the tumor region with 10 and 250 Hz of PRF.

The cavitation behavior of IMP700 was evaluated in the PANC-1 xenograft model using different duty cycles (1–5%) and PRFs (10 and 250 Hz). Stable cavitation increased with rising duty cycle in both the nanodroplet and FUS-only groups (Figure 8a). In the IMP700 group, the stable cavitation dose increased from 0.58 V^2^s at 1% duty cycle to 6.71 V^2^s at 5%, showing a comparable increasing trend to that of the FUS-only group. By contrast, inertial cavitation exhibited a markedly different pattern depending on the presence of IMP700 (Figure 8b). At a duty cycle of 1%, tumors treated with IMP700 generated an inertial cavitation dose of 14.19 V^2^s, whereas FUS-only tumors showed only 1.18 V^2^s, which demonstrates a pronounced amplification of inertial cavitation by IMP700. This substantial difference persisted across higher duty cycles, reaching 30.92 V^2^s in the IMP700 group compared to 2.42 V^2^s in the FUS-only group at a 5% duty cycle. Although inertial cavitation increased with duty cycle, no further enhancement beyond the peak response was observed, which suggests saturation of cavitation activity at higher duty cycles.

At a fixed duty cycle, 10 Hz PRF resulted in considerably higher stable and inertial cavitation compared to 250 Hz in both the IMP700-treated and FUS-only groups (Figure 8c,d). In the IMP700 group, stable cavitation decreased from 1.95 V^2^s at 10 Hz to 0.1 V^2^s at 250 Hz, and inertial cavitation decreased from 13.62 to 0.89 V^2^s, which indicates that lower PRFs strongly promote cavitation activity in vivo. Based on these measurements, a duty cycle of 2% and a PRF of 10 Hz were used for subsequent sonoporation experiments.

### 3.9. Sonoporation Efficiency

**Figure 9:**
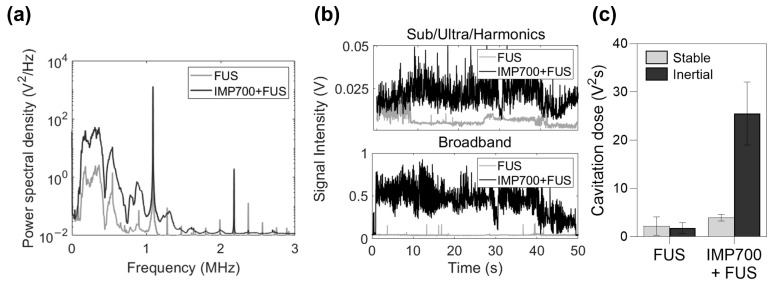
In vivo cavitation enhancement and sonoporation in PANC-1 tumor. (**a**) Frequency- and (**b**) time domain spectra with or without IMP700. (**c**) Quantitative comparison of stable and inertial cavitation doses.

Cavitation activity was evaluated in PANC-1 tumors following intravenous administration of IMP301 with or without IMP700 and subsequent FUS exposure (2 kW/cm^2^, 2% duty cycle, and 10 Hz PRF) (Figure 9a–c). In the frequency-domain spectra, tumors treated with IMP301 + IMP700 + FUS exhibited higher broadband and harmonic signals compared to the IMP301 + FUS group (Figure 9a). Time-domain analysis revealed increasing acoustic signal intensity over time when IMP700 was co-administered, while the IMP301 + FUS group exhibited only minimal activity (Figure 9b). Quantitative cavitation analysis showed that the inertial cavitation dose was substantially higher in the IMP700-treated group, whereas the stable cavitation remained low and comparable between both groups (Figure 9c).

**Figure 10:**
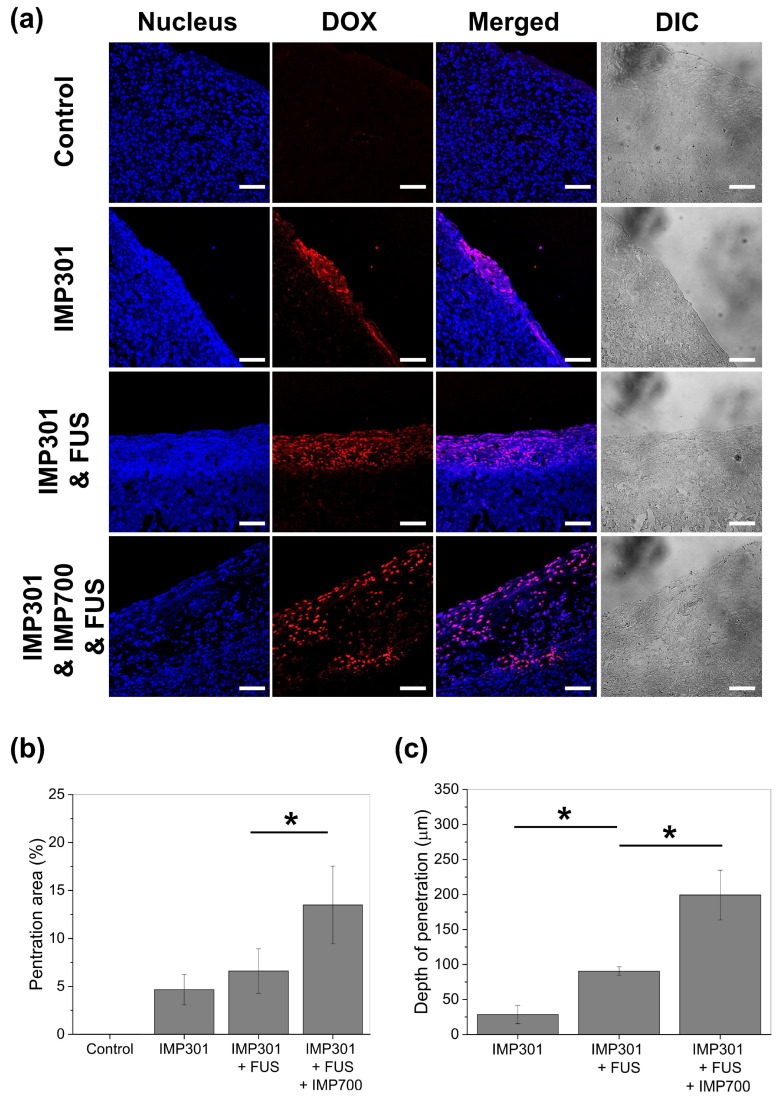
Sonoporation efficiency of DOX to tumor region under the FUS irradiation at 2.0 kW/cm^2^ of intensity, 2% of duty cycle, and 10 Hz of PRF. (**a**) Fluorescence image of tumor region stained by DAPI (blue) and DOX (red). Scale bar: 100 μm. Quantitative results of (**b**) penetration area and (**c**) depth from tumor margin. * *p* < 0.05.

Following cavitation monitoring, tumor tissues were harvested one hour after FUS exposure and examined by confocal fluorescence imaging to assess the sonoporation effects (Figure 10a). In the IMP301 group, DOX fluorescence was mainly restricted to the tumor periphery, with little penetration beneath the tumor surface. When combined with FUS, the group showed an increased DOX signal with deeper penetration from the tumor surface compared to IMP301 alone. Co-administration of IMP700 and IMP301 followed by FUS resulted in DOX fluorescence penetrating more deeply into the tumor and exhibiting the broadest distribution across the tissue section.

Quantitative analysis showed progressive increases in both penetration area (Figure 10b) and penetration depth (Figure 10c and Figure S4). The DOX-positive area was about 5% in the IMP301 group and increased slightly in the IMP301 + FUS group. The IMP301 + IMP700 + FUS group showed the highest penetration area, with approximately a threefold increase compared to the IMP301 group. The DOX penetration depth was less than 30 µm in the IMP301 group and increased to approximately 90 µm when combined with FUS. With IMP700 and FUS, the penetration depth further increased to about 200 µm, reaching more than six times that of the IMP301 group. These results demonstrate that IMP700 enhances sonoporation efficiency by increasing the extent and depth of IMP301 delivery within tumors. Consistently, TUNEL staining of PANC-1 tumor sections demonstrated increased apoptotic regions in the IMP301 + IMP700 + FUS group, showing a similar trend to the enhanced drug penetration observed above (Figure S5).

### 3.10. Enhancement of CD8^+^ T-Cell Infiltration to Tumor via IMP700 Vaporization

**Figure 11:**
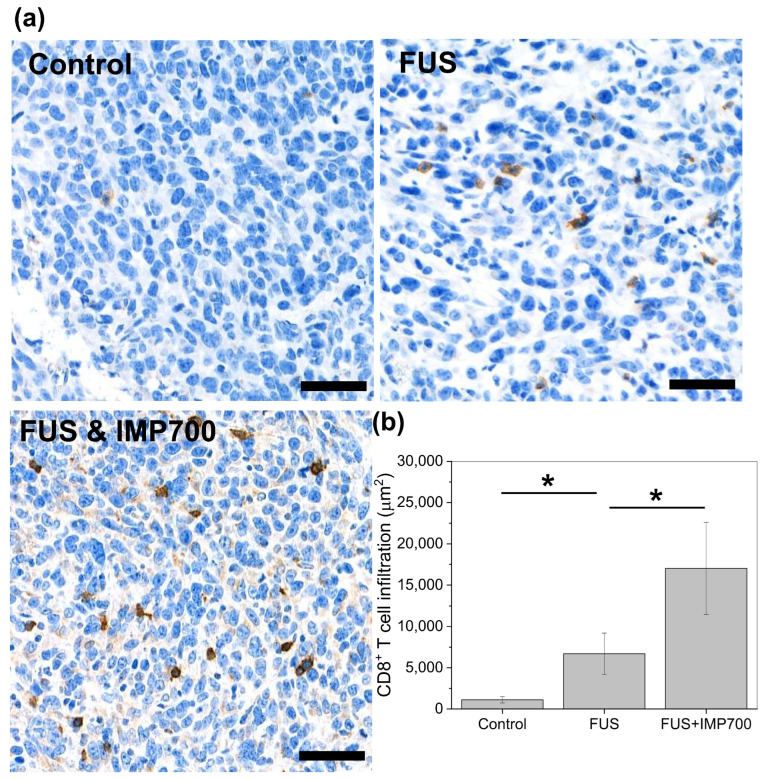
CD8^+^ T-cell infiltration in tumor region in 4T1 syngeneic model. (**a**) IHC image stained by CD8α antibody (brown color). (**b**) Quantitative image of CD8α-stained spot. Scale bar: 50 μm. Statistical analysis was performed using the Kruskal–Wallis test with Dunn’s post hoc test; * *p* < 0.05.

To evaluate the immune response enhancement induced by the combination of FUS and IMP700, CD8^+^ T-cell infiltration into tumors was assessed by immunohistochemistry. Representative CD8α-stained tumor sections from each group are shown in Figure 11a. In tumors without FUS treatment, CD8^+^ T-cell infiltration was rarely observed. By contrast, increased numbers of CD8^+^ T cells were detected in the tumor regions of both the FUS and FUS + IMP700 groups. Notably, the FUS + IMP700 group exhibited the most pronounced CD8^+^ T-cell infiltration among all experimental groups. Lower-magnification images illustrating the overall distribution of CD8^+^ T cells across tumor sections are provided in Supplementary Figure S6.

The quantitative analysis of CD8^+^ T-cell infiltration is presented in Figure 11b. The DAB-positive area in the control, FUS, and FUS + IMP700 groups was 1111.00 ± 370.85, 6704.06 ± 2499.78, and 17027.40 ± 5577.04 µm^2^, respectively. These results demonstrate that FUS treatment alone enhanced CD8^+^ T-cell infiltration, while the addition of IMP700 further potentiated this effect. Collectively, these findings suggest that cavitation plays a key role in FUS-mediated immune activation and that IMP700 amplifies this response through acoustic vaporization.

## 4. Discussion

This study successfully develops and characterizes PFP-based nanodroplets (IMP700) as ultrasound-responsive agents for enhancing cavitation, intratumoral drug delivery, and antitumor immune responses in the solid tumor. Our results show that IMP700 undergoes efficient ADV under FUS, significantly amplifies inertial cavitation, and substantially improves ultrasound-mediated drug penetration in dense tumor tissue. Importantly, combined IMP700 and FUS treatment also increased intratumoral CD8^+^ T-cell infiltration, which highlights the potential of FUS-activated nanodroplets to overcome both stromal and immunological barriers in solid tumors.

### 4.1. Physicochemical Properties and Stability of IMP700

The IMP700 exhibited a Z-average diameter of 326 ± 64.7 nm, a size range in which extravasation through leaky tumor vasculature has been reported. Furthermore, the particle size was maintained in serum-containing culture medium, which indicates that the formulation is expected to remain stable under physiological vascular conditions (Figure S2). While ~300 nm is modestly larger than the size range typically associated with efficient EPR-mediated transport (~200 nm), cavitation-mediated vascular permeabilization is expected to promote active extravasation. [28,29]. In addition, this nanoscale size enables more effective sonoporation within tumor tissue compared to conventional microbubbles. This size range of IMP700 provides a clear advantage over conventional microbubbles (>1 µm), which remain primarily within the bloodstream and show minimal penetration into tumor tissue [30,31]. The high encapsulation efficiency of PFP (99.5%) confirms the successful incorporation of the PFP core, which is an essential feature enabling effective activation by ultrasound.

Cryo-TEM imaging provided direct morphological evidence of ADV. Prior to ultrasound exposure, IMP700 maintained a uniform spherical structure, whereas post-vaporization images displayed disrupted, porous morphologies consistent with gas bubble formation and shell fragmentation. This phase transition is the fundamental mechanism by which nanodroplets generate the localized cavitation effects necessary for sonoporation [21,32]. Our stability studies revealed that IMP700 concentration maintained over 24 h at 4 °C, with particle counts and stable size distribution. Diffusion of C_5_F_12_ within IMP700 is not observed, and vaporization is suppressed due to the elevated boiling point resulting from the Laplace pressure imposed by the shell [33]. Temperature-dependent microscopy further demonstrated that IMP700 is thermally sensitive, with spontaneous vaporization occurring at 37 °C and complete phase transition at 60 °C. These findings indicate that IMP700 should be stored at low temperatures and used without delay to maintain stability.

### 4.2. Acoustic Droplet Vaporization and Cavitation Behavior In Vitro

Our in vitro experiments confirmed that IMP700 undergoes intensity-dependent ADV. Echogenicity increased progressively with ultrasound intensity from 0.35 to 2.81 kW/cm^2^, which demonstrates that FUS can trigger the liquid-to-gas phase transition of IMP700s. This behavior is consistent with prior observations that perfluorocarbon droplets exhibit vaporization above a certain intensity [33,34].

The cavitation behavior of IMP700 vaporization was investigated under different FUS intensities. The FUS intensity was selectively varied while keeping the duty cycle and PRF constant. IMP700 incorporates a PFP core, and its vaporization is predominantly regulated by acoustic pressure (ultrasound intensity), which serves as the key trigger for ultrasound-induced phase transition [21,35]. To specifically assess the influence of acoustic pressure on droplet vaporization, the duty cycle and PRF were fixed at 2% and 10 Hz, respectively, as temporal parameters will be systematically optimized in subsequent in vivo cavitation studies.

Cavitation analysis showed that the stable cavitation dose increased steadily from 1.0 to 2.5 kW/cm^2^, whereas inertial cavitation rose sharply at intensities ≥ 2.0 kW/cm^2^. Stable cavitation, characterized by sustained bubble oscillations, is typically associated with reversible membrane permeabilization and modest enhancement of vascular permeability [36]. By contrast, inertial cavitation results from violent bubble collapse and generates shock waves and microjets that can disrupt the extracellular matrix and produce transient pores in cell membranes [37,38]. Although these effects can be advantageous for improving drug penetration in fibrotic tumors, excessive inertial cavitation may induce microvascular injury or hemorrhage, ultimately limiting therapeutic delivery [39,40].

Based on these experimental observations, we selected 2.0 kW/cm^2^ as the experimental intensity for subsequent in vivo studies. Although the exact cavitation thresholds cannot be fully defined from the current data alone, this setting consistently produced sufficient inertial cavitation to support sonoporation without eliciting excessive acoustic effects.

### 4.3. In Vivo Cavitation and Optimization of Ultrasound Parameters

In vivo cavitation studies in the PANC-1 xenograft model demonstrated that duty cycle and PRF significantly influence cavitation behavior following intravenous IMP700 injections. Specifically, stable cavitation increased with duty cycle from 1% to 5%, whereas inertial cavitation did not increase proportionally with duty cycle in the presence of IMP700 and showed an apparent plateau beyond 2%. One possible explanation is that, at the given IMP700 dose, the number of droplets that can participate in cavitation is already saturated at low duty cycles. Increasing the duty cycle further mainly causes repeated collapse of the same bubbles rather than involving additional droplets, so inertial cavitation no longer increases. In addition, the dense, fibrotic stroma of PDAC is known to constrain transport and modulate the local mechanical environment [41,42], which may further restrict bubble motion and attenuate inertial cavitation despite higher acoustic exposure. A duty cycle of 2% was, therefore, selected for subsequent sonoporation experiments, as it represented the lowest setting that elicited a marked increase in inertial cavitation compared to 1% while maintaining cavitation enhancement, without indications of excessive damage at higher duty cycles.

Notably, PRF had an even stronger influence on cavitation activity. A PRF of 10 Hz generated markedly higher stable and inertial cavitation compared to 250 Hz, both with and without nanodroplets. A likely explanation is that, at lower PRF, the longer interval between pulses allows new nanodroplets and microbubbles to flow into the focal region. Previously formed bubbles also have time to dissipate, so each pulse interacts with a refreshed population of bubbles. At very high PRF, pulses act on the same bubbles in rapid sequence, which leads to their fast destruction and loss before they can be replaced. As a result, the overall cavitation strength is reduced even though the pulse rate is higher. Similar PRF-dependent effects on cavitation and drug delivery have been reported in several studies [43,44]. However, only two PRF conditions (10 and 250 Hz) were tested in this study, which is a limitation that does not allow detailed evaluation of how PRF affects cavitation behavior. Within this limitation, 10 Hz was selected as the PRF for subsequent sonoporation experiments, as it yielded the strongest and most reproducible cavitation under our experimental conditions.

### 4.4. Enhanced Sonoporation and Intratumoral Drug Delivery

The present study demonstrates that IMP700 effectively enhances the intratumoral delivery of IMP301 under FUS exposure by amplifying cavitation activity within the pancreatic tumor microenvironment. Confocal imaging showed clear differences in the distribution of doxorubicin, with the IMP700-treated group exhibiting the deepest and broadest penetration among all conditions.

Pancreatic ductal adenocarcinoma is characterized by a dense and fibrotic stroma that restricts the transport of therapeutic agents, which frequently diminishes the efficacy of nanoparticle-based drug delivery [45,46,47]. Within this challenging microenvironment, the improved distribution of doxorubicin achieved with IMP700 is likely attributable to cavitation-induced mechanical effects. ADV produces localized microbubbles that undergo inertial cavitation, generating mechanical forces that transiently loosen stromal structures and enhance interstitial porosity [48]. Cavitation also helps particles move through regions with poor permeability, and the transient increase in membrane permeability facilitates cellular uptake [49]. Collectively, these processes create enhanced tissue permeability that enables deeper intratumoral penetration.

Importantly, the cavitation measurements confirmed that IMP700 substantially amplified inertial cavitation in vivo, whereas IMP301 + FUS alone produced minimal cavitation activity. These findings support cavitation as the principal mechanism driving improved doxorubicin delivery. Although the increase in cavitation was not strictly proportional across all ultrasound settings, the cavitation threshold achieved with IMP700 was sufficient to meaningfully modify tissue permeability and facilitate deeper penetration of the co-administered therapeutic payload.

Overall, these findings highlight the therapeutic potential of IMP700 as an ultrasound-responsive agent that can modulate the physical barriers of pancreatic tumors. By enhancing cavitation dynamics within the tumor, IMP700 improves the penetration of nanoparticle therapeutics such as IMP301, which suggests a broadly applicable strategy for addressing the stromal resistance that limits drug efficacy in pancreatic cancer.

### 4.5. Amplification of the Immune Response by IMP700 Cavitation

Focused ultrasound (FUS) has emerged as a promising non-invasive modality capable of modulating the tumor microenvironment through mechanical effects, including acoustic cavitation. In the present study, we demonstrate that FUS treatment enhances the intratumoral infiltration of CD8^+^ T-cells and that this immunomodulatory effect is further potentiated by the vaporization-capable agent IMP700.

Immunohistochemical analysis revealed minimal CD8^+^ T-cell infiltration in untreated tumors, whereas FUS treatment alone increased CD8^+^ T-cell presence within the tumor microenvironment. This observation is consistent with previous reports showing that cavitation induced by ultrasound can transiently disrupt tumor vasculature, increase vascular permeability, and facilitate immune cell recruitment into solid tumors [1,2,3]. Such mechanical perturbations are known to promote antigen release and enhance immune surveillance, thereby contributing to antitumor immune activation.

Notably, the combination of FUS with IMP700 resulted in a marked and synergistic increase in CD8^+^ T-cell infiltration compared to FUS treatment alone. This enhanced effect is likely attributable to the acoustic vaporization of IMP700, which amplifies cavitation activity under FUS exposure. Phase-transition agents capable of vaporization have been reported to significantly lower cavitation thresholds and intensify mechanical stresses within tumor tissue, leading to more pronounced biological effects [20,32]. In this context, IMP700 appears to function as an effective cavitation enhancer, thereby augmenting the immunological consequences of FUS treatment.

The increased infiltration of CD8^+^ T cells observed in the FUS + IMP700 group suggests a shift toward a more immunologically active tumor microenvironment. Cytotoxic CD8^+^ T cells play a central role in antitumor immunity by directly eliminating malignant cells and orchestrating downstream immune responses [50]. Therefore, the substantial increase in the CD8^+^ T-cell-positive area following combined treatment indicates that cavitation-enhanced FUS may serve as a potent immune-priming strategy. Collectively, these findings provide compelling evidence that vaporization-mediated cavitation amplification using IMP700 significantly enhances the immunomodulatory effects of FUS, supporting its potential as a combinatorial strategy for cancer immunotherapy. In our previous study, cavitation by FUS demonstrated immunogenic cell death with the increase in inflammation cytokines and with the modulation of the tumor microenvironment [51]. FUS monotherapy enhanced immune activation such as M1/M2 ratio, NK cells, and CD4 and CD8 T-cell-resulting promotion of tumor cell death. According to previous results, the combination of IMP700 and FUS is sufficiently capable for the enhancement of immunogenic cell death by the amplification of cavitation via vaporization of C_5_F_12_. Therefore, more detailed investigations into the immunotherapeutic implications of this approach are warranted and will be pursued in future studies.

### 4.6. Limitations and Future Directions

Several limitations of this study should be noted. First, IMP700 demonstrated limited stability over 24 h, which indicates the need for improved formulations with enhanced storage stability. Second, while this study demonstrated enhanced drug penetration using doxorubicin as a model payload, the therapeutic efficacy of IMP700-mediated delivery remains to be determined. Future studies should, therefore, evaluate treatment outcomes including tumor regression, survival, and potential synergy with immune checkpoint inhibitors or other combination strategies.

In addition, the potential safety implications of increasing tumor permeability should be considered. Although excessive permeability could theoretically increase susceptibility to pathogen entry [52], the optimized FUS parameters used in this study induced localized and transient cavitation confined to the focal tumor region. Unlike approaches targeting critical physiological barriers such as the blood–brain barrier, our strategy focuses on tumor tissue with already abnormal vasculature. While further safety evaluation is warranted, our findings suggest that FUS-activated nanodroplets enhance permeability primarily within tumor tissue, with a low likelihood of unintended systemic effects.

Finally, the present study focused on CD8^+^ T-cell infiltration as a key indicator of immune activation. Further investigations are warranted to elucidate additional immunological mechanisms, including antigen presentation, cytokine release, and potential synergy with immune checkpoint blockade [53,54].

## 5. Conclusions

In this study, we developed and evaluated a PFP-based phase-change nanodroplet, IMP700, as an ultrasound-responsive agent to enhance cavitation, intratumoral drug delivery, and immune engagement in solid tumors. IMP700 exhibited a nanoscale size suitable for tumor extravasation, high PFP encapsulation efficiency, and clear ADV under FUS exposure. Systematic in vitro and in vivo cavitation assessments enabled the identification of ultrasound parameters that robustly generated inertial cavitation in vivo, with 2 kW/cm^2^ intensity, 2% duty cycle, and 10 Hz PRF selected for subsequent experiments.

Under these conditions, co-administration of IMP700 with an ultrasound-responsive liposomal formulation (IMP301) significantly increased inertial cavitation activity and promoted deeper, broader intratumoral penetration of the drug payload. These findings indicate that nanodroplet-mediated cavitation effectively modulates the physical barriers of solid tumors, facilitating deeper transport of therapeutic payloads. Importantly, in a syngeneic 4T1 solid tumor model, IMP700-assisted FUS treatment led to a pronounced increase in intratumoral CD8^+^ T-cell infiltration compared to FUS alone, supporting a role for vaporization-mediated cavitation amplification in enhancing the immunomodulatory effects of FUS.

Overall, our study establishes IMP700 as a versatile cavitation-enhancing agent that strengthens both the drug-delivery and immunomodulatory effects of FUS. This nanodroplet-assisted FUS strategy offers a broadly applicable physical platform for overcoming stromal and immunological barriers in solid tumors and provides a foundation for future therapeutic combinations integrating ultrasound with immunotherapy or nanomedicine.

## Figures and Tables

**Table 1:** Summary of frequency bands used for stable and inertial cavitation analysis.

	Harmonic Type	Frequency Range (MHz)
Fundamental frequency	-	1.09–1.11
Stable cavitation bands	Subharmonic	0.355–0.375, 0.54–0.56
	Ultraharmonic	1.64–1.66, 2.74–2.76
	Harmonic	2.19–2.21
Inertial cavitation bands		0.2–0.355, 0.375–0.54, 0.56–1.09, 1.11–1.64, 1.66–2.19, 2.21–2.74, 2.76–3

## Data Availability

The data presented in this study are available on reasonable request from the corresponding author.

## Supplementary Materials

The following supporting information can be downloaded at: https://www.mdpi.com/article/10.3390/pharmaceutics18030302/s1, Figure S1: The characteristics of IMP301; Figure S2: Size characterization and media stability of IMP700; Figure S3: (a) B-mode US image and enhanced (b) gray scale of IMP700 vaporization in tumor region at pre and post FUS irradiation; Figure S4: Cross-sectional fluorescence image of doxorubicin penetration to the tumor region; Figure S5: Representative paraffin-embedded PANC-1 tumor sections stained by TUNEL assay for the indicated treatment groups; Figure S6: Immunohistochemical staining of CD8^+^ T cells in tumor sections from the Control, FUS, and FUS + IMP700 groups.
