# Supplementary material for: Perfluoropentane Phase-Change Nanodroplets for Focused Ultrasound-Enhanced Drug Penetration and Immune Response
Source: Pharmaceutics. 2026 Feb 27;18(3):302. doi: 10.3390/pharmaceutics18030302 (PMC13029623; doi:10.3390/pharmaceutics18030302)
Supplement: Supplementary file 1 [file pharmaceutics-18-00302-s001.zip › pharmaceutics-4124399-supplementary.pdf]

Supplementary materials

Perfluoropentane Phase-Change Nanodroplets for Focused Ultrasound-Enhanced Drug Penetration and Immune Response

Kichang Shin [1], Dongyeon Kim [2], Hyungwon Moon [3], Keunho Son [3], Mi Jeong Kim [4] and Hak Jong Lee [1,2,3,4,*]

[1] Department of Health Science and Technology, Graduate School of Convergence Science and Technology, Seoul National University, 1 Gwanak-ro, Gwanak-gu, Seoul 08826, Republic of Korea; skc5027@snu.ac.kr

[2] Department of Applied Bioengineering, Graduate School of Convergence Science and Technology, Seoul National University, 1 Gwanak-ro, Gwanak-gu, Seoul 08826, Republic of Korea; dongyeonk619@snu.ac.kr

[3] R&D Center, IMGT Co., Ltd., 172, Dolma-ro, Bundang-gu, Seongnam-si 13605, Republic of Korea; hyungwon.moon@nanoimgt.com (H.M.); keonho.son@nanoimgt.com (K.S.)

[4] Department of Radiology, Seoul National University Bundang Hospital, Seongnam-si 13620, Republic of Korea; mijeong.kim@nanoimgt.com

* Correspondence: hakjlee@snu.ac.kr

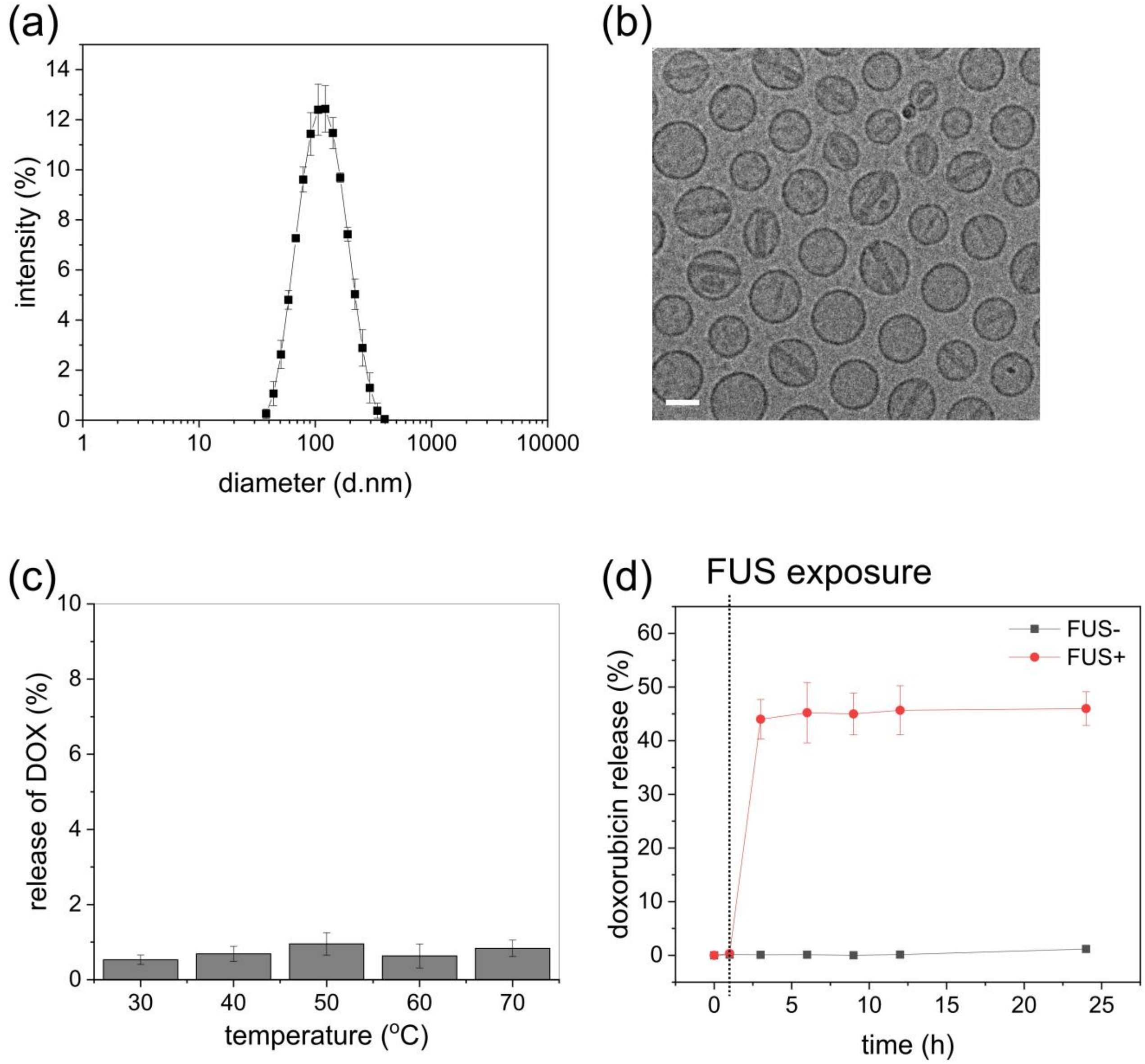

Figure S1. The characteristics of IMP301. (**a**) size distribution, (**b**) cryo-TEM image, (**c**) release ratio of doxorubicin (DOX) dependent on the temperature, (**d**) in vitro release after FUS irradiation at 0.5h., Encapsulation efficiency was 97.1 ± 1.44%.

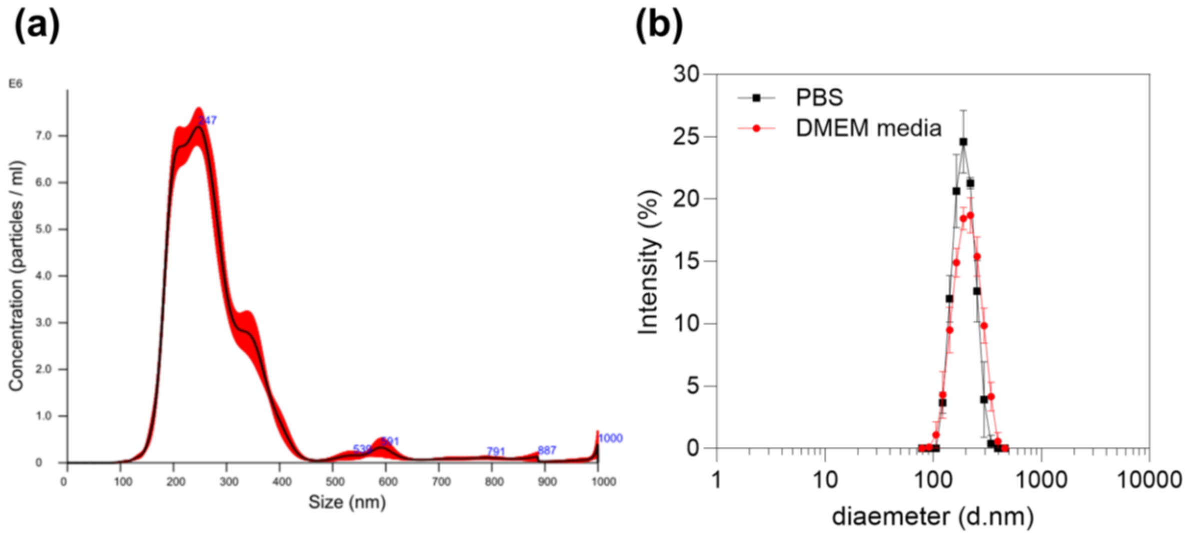

Figure S2. Size characterization and media stability of IMP700. (**a**) NTA showing the particle size distribution of IMP700 with a mean diameter of 285.2 nm. (**b**) DLS size distribution of IMP700 measured in PBS and DMEM, demonstrating hydrodynamic diameters and stability in DMEM medium.

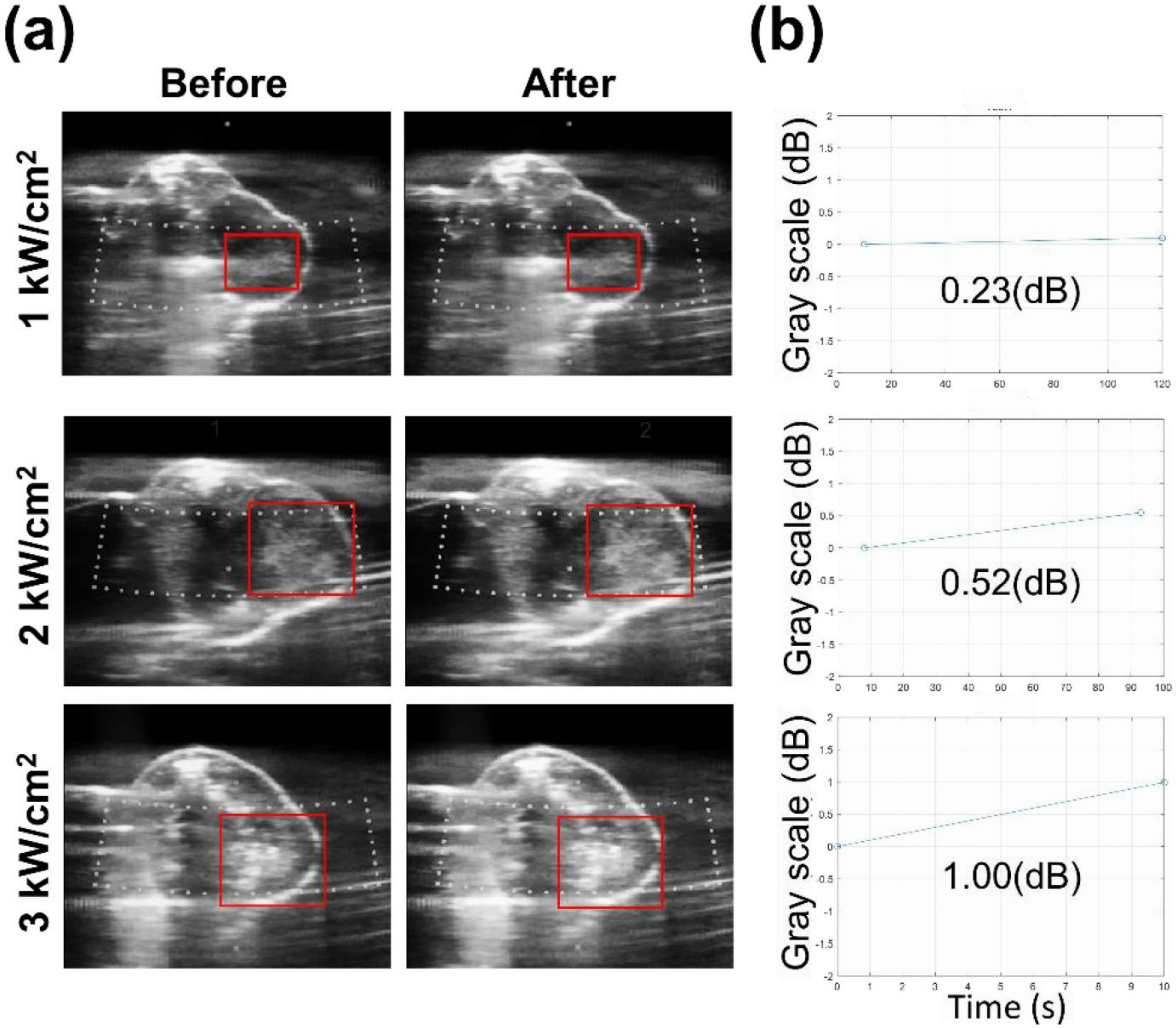

Figure S3. (**a**) B-mode US image and enhanced (**b**) gray scale of IMP700 vaporization in tumor region at pre and post FUS irradiation.

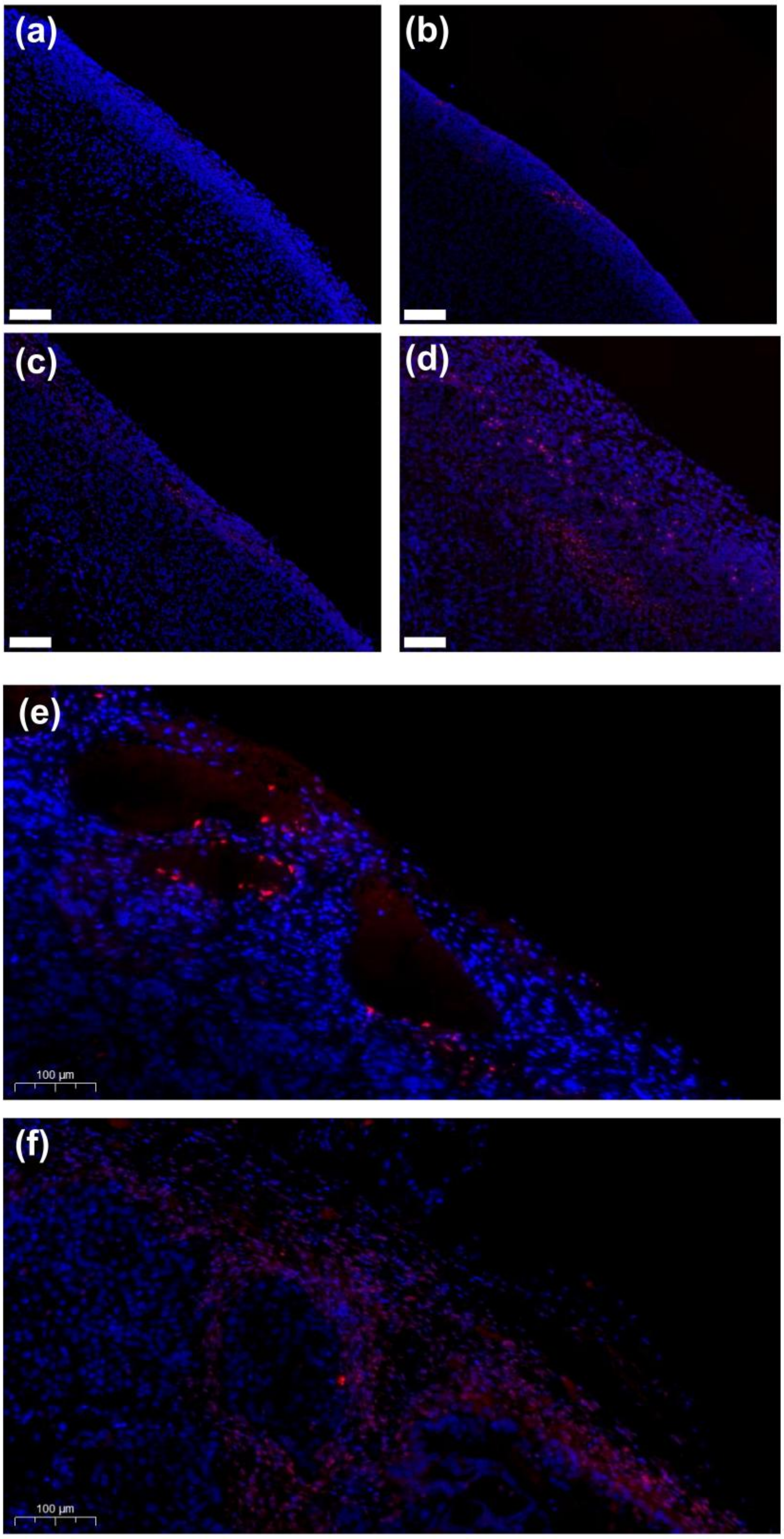

Figure S4. Cross-sectional fluorescence image of doxorubicin penetration to the tumor region. (**a**) negative control group, (**b**) IMP301 group, (**c**) IMP301 with FUS exposure group and (**d**) IMP301 and IMP700 with the FUS exposure group. and magnificent image of IMP301 and IMP700 (**e**) without and (**f**) with FUS exposure. Scale bar; 100 µm.

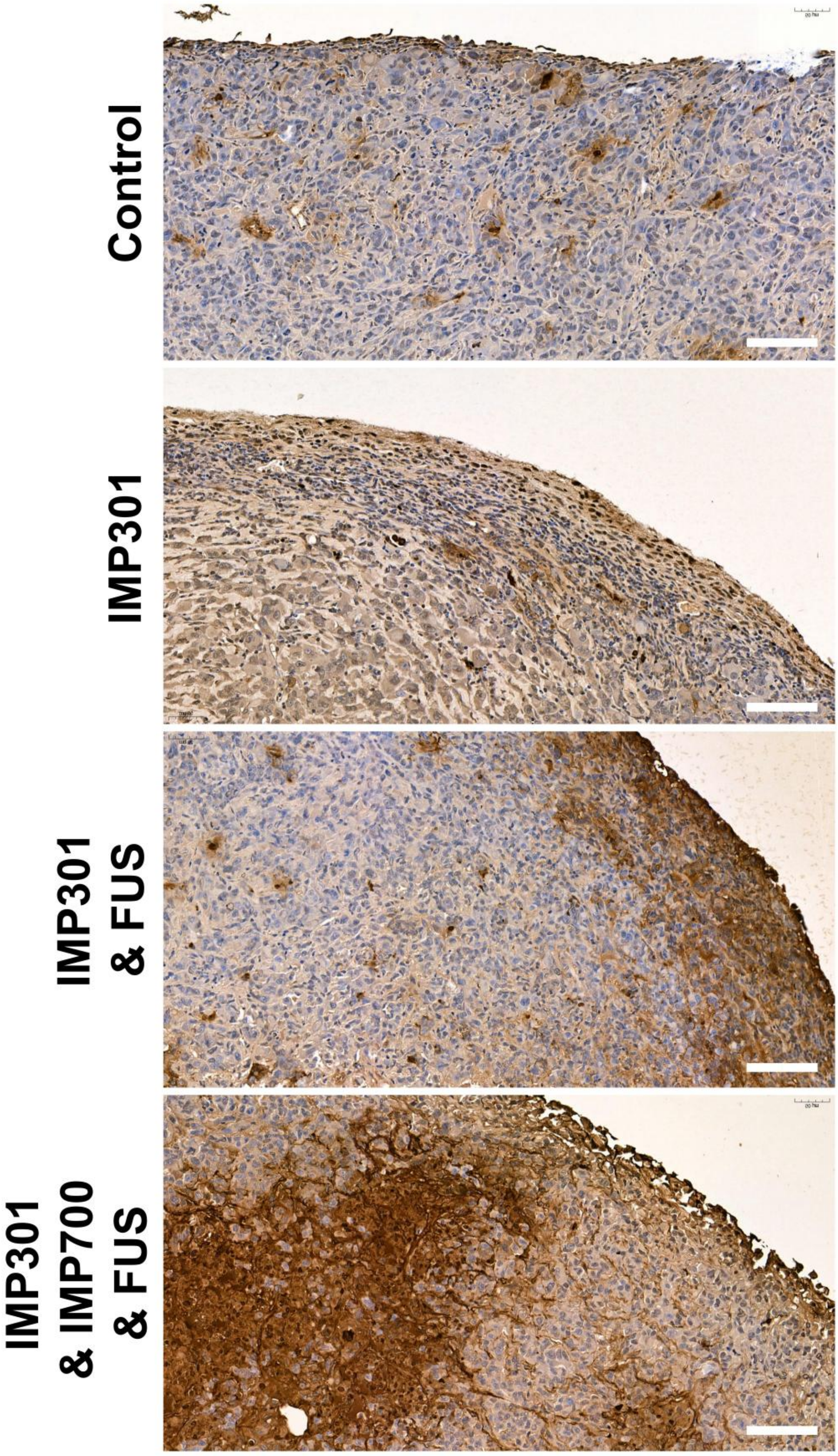

Figure S5. Representative paraffin-embedded PANC-1 tumor sections stained by TUNEL assay for the indicated treatment groups. The IMP301 + IMP700 + FUS group exhibited increased apoptotic regions compared with the other groups, consistent with the trend observed in Figure 10a. Scale bars, 100 μm.

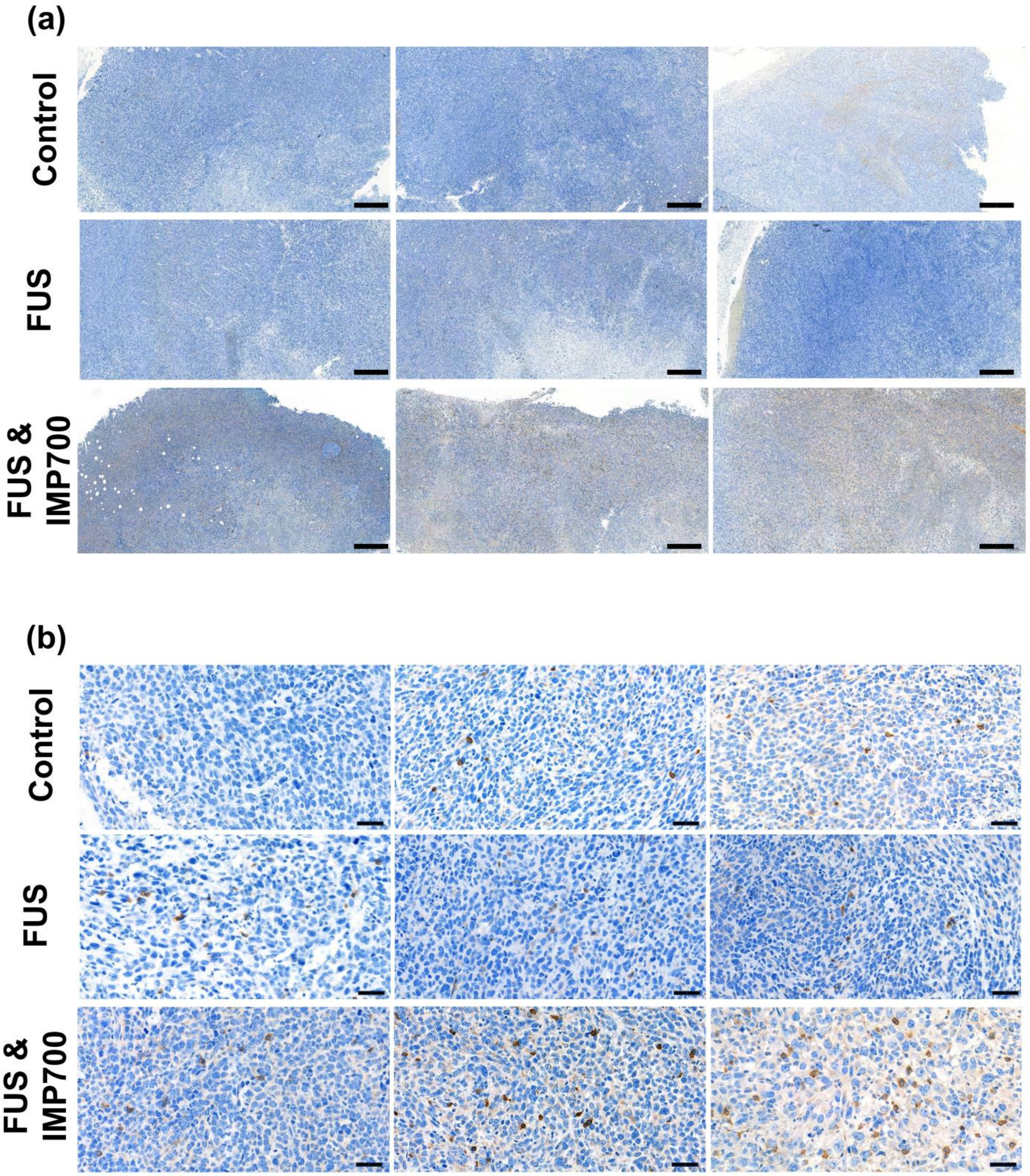

Figure S6. Immunohistochemical staining of $CD8^+$ T cells in tumor sections from the Control, FUS, and FUS + IMP700 groups. (**a**) low magnification (4x) and (**b**) high magnification (20x) Whole-tumor sections are shown at low magnification Scale bars: low magnification(black), 200 μm, high magnification(white) 40 μm.
